# Methyl-β-cyclodextrin up-regulates collagen I expression in chronologically-aged skin via its anti-caveolin-1 activity

**DOI:** 10.18632/oncotarget.3039

**Published:** 2014-12-10

**Authors:** Jung-Ae Lee, Da-In Choi, Jee-Young Choi, Sun-Ok Kim, Kyung-A Cho, Jee-Bum Lee, Sook-Jung Yun, Seung-Chul Lee

**Affiliations:** ^1^ Department of Dermatology, Chonnam National University Medical School, Gwangju, Korea; ^2^ Department of Urology, Chonnam National University Medical School, Gwangju, Korea; ^3^ Department of Biochemistry, Chonnam National University Medical School, Gwangju, Korea

**Keywords:** Anti-aging, Caveolin-1, Collagen I, Methyl-β-cyclodextrin

## Abstract

Caveolin-1 (Cav-1) is one of the key molecules to modulate collagen metabolism in the skin. This study aimed to unravel the relationship between Cav-1 and collagen levels in the aged skin, and also to evaluate a new role of anti-Cav-1 agent as a collagen-modulating agent. A negative correlation between Cav-1 and collagen I (COL I) was detected in chronologically aged skin of humans and mice, which was further confirmed by Cav-1 knock-down or knock-out experiments. Next, we tested whether methyl-β-cyclodextrin (MβCD) as a chemical Cav-1 inhibitor could be developed as a collagen-modulating agent in the skin. Testing different conditions of MβCD injection via the intra-dermal route revealed that 2.5% MβCD administered twice per week for two months showed a potent COL I-up-regulating activity, leading to the increase of skin thickness (*P* < 0.05) without adverse reactions such as skin fibrosis. In human dermal fibroblasts, MβCD treatment induced up-regulated COL I and down-regulated Cav-1, supporting the results of mouse experiments. Collectively, MβCD has a COL I-enhancing activity in chronologically-aged skin, where Cav-1 acts as a brake in COL I expression, suggesting its potential role for an anti-aging agent.

## INTRODUCTION

Caveolins are principal integral membrane proteins of caveolae, which comprise at least four proteins – caveolin-1α, 1β (21–24 kDa), and −2 (20 kDa) – that are co-expressed to form hetero-oligomeric complex in many cells, whereas caveolin-3 (18 kDa) is muscle specific [1-3]. Besides their structural role, caveolins have a gate-keeping role in modulating various signaling molecules localized to the membrane component [4, 5]. Interestingly, Cav-1 was reported to play a critical role in the promotion of cellular senescence [6, 7]. Cav-1 was increased in senescent dermal fibroblasts (DFs), and over-expression of Cav-1 induced premature cellular aging [8, 9]. Despite evidence showing the crucial role of Cav-1 in cellular senescence, few studies have been performed on the age-dependent change of Cav-1 in the human and mouse skin *in vivo*.

In the skin, Cav-1 has been implicated in pathological conditions of abnormal collagen expression. Cav-1 level was decreased in the skin of scleroderma or keloid, characterized by up-regulated expression of dermal collagens [10-12]. Cav-1 was reported to exert an inhibitory activity by modulating transforming growth factor-β (TGF-β) signaling through its participation in TGF-β receptor (TβR) internalization [10]. These studies suggest that Cav-1 plays a brake role in collagen expression in the skin or DFs. In contrast, Cav-1 was reported to play an accelerator role by up-regulating collagen I (COL I) via the TGF-β pathway in DFs or Cav-1 transgenic mice [13, 14]. The conflicting results concerning the expressional relationship between Cav-1 and collagens in the skin prompted us to evaluate Cav-1 level in chronologically-aged skin, which is supposed to be an ideal model to unravel the relationship between their expression levels. In relation with skin aging, reduced TGF-β/TβRs signaling is supposed to be critical for the loss of COL I expression in chronologically-aged skin [15]. Among the collagen families, COL I is the major component to determine the total bulk of collagens and tensile strength of the skin. With these backgrounds, we focused on the modulation of COL I in studying a new candidate molecule to modulate collagen levels in the skin.

Methyl-β-cyclodexrin (MβCD), which is synthesized by random methylation of β-cyclodextrin (-CD), is a cholesterol-depleting agent that disrupts caveolae by virtue of anti-Cav-1 activity [16]. CDs have been widely used for foods, in drug delivery, in cosmetics, and in chemicals, as well as agriculture and environmental engineering, as they are very safe molecules to our body [17]. The most common CDs are −α-CD, β-CD, and γ-CD, having six, seven, and eight anhydroglucose units in their ring structure, respectively [18, 19]. This study evaluated whether MβCD has a potential to modulate COL I level by its anti-Cav-1 activity in the skin, especially chronologically-aged skin.

## RESULTS

### Cav-1 is up-regulated, but COL I is down-regulated, in chronologically-aged skin of human and mouse

**Figure 1:**
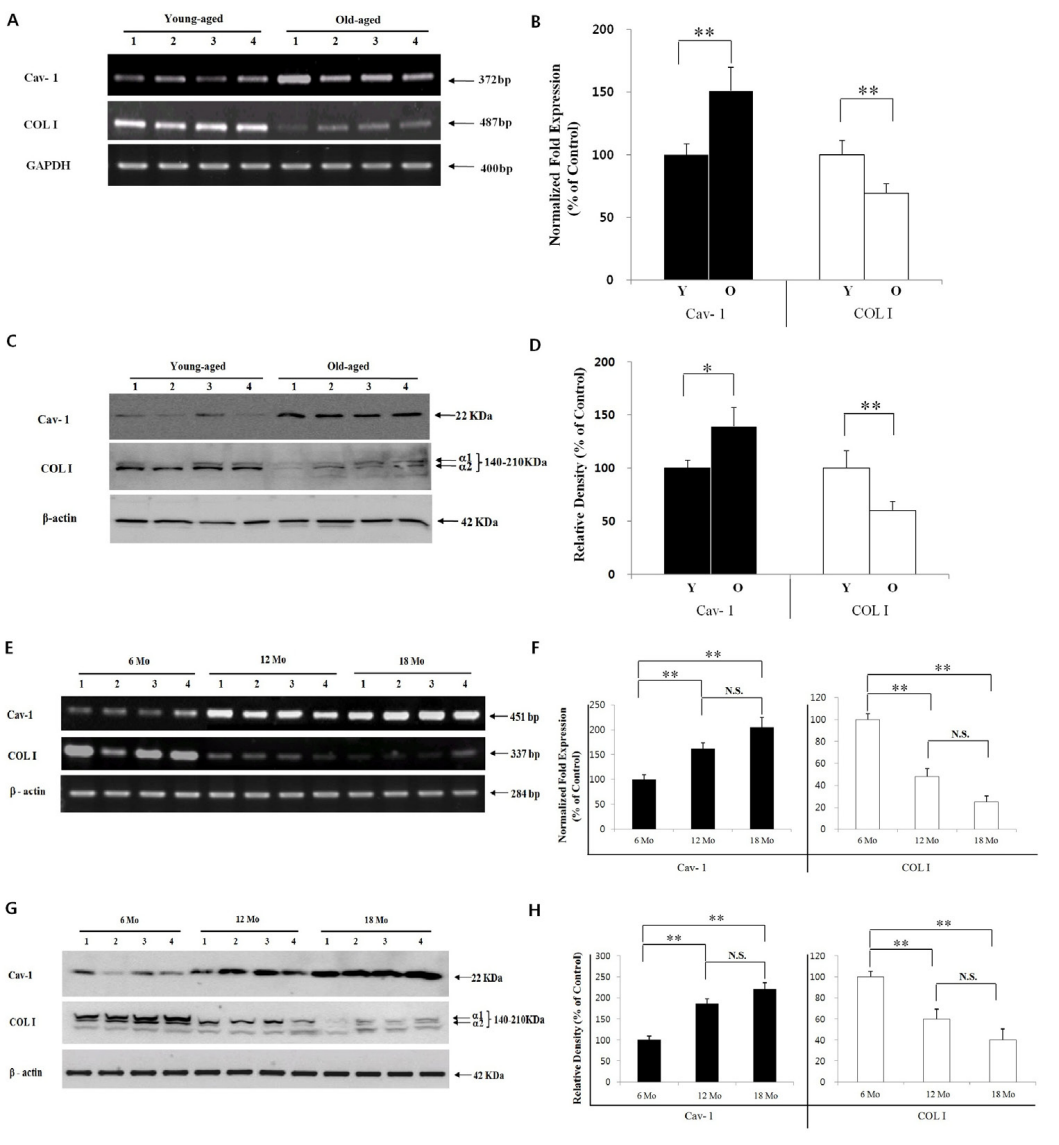
Cav-1 expression is negatively related with COL I expression in chronologically-aged human and mouse skin To compare the age-dependent change of Cav-1 and COL I, skin tissues were excised from the young humans (Y, n=4) and more elderly persons (O, n=4). (A) RT-PCR, (B) real time-PCR, and (C) Western blot analysis followed by densitometric analysis (D) of Cav-1 and COL I proteins in the skin was performed. Next, age-dependent changes of mRNA levels checked by (E) RT-PCR and (F) real-time PCR, and protein levels checked by (G) Western blot analysis followed by densitometric analysis (H) of Cav-1 and COL I proteins were also studied in hairless mice from different age-groups of 6-months-old (6 Mo), 12-months-old (12 Mo), and 18-months-old (18 Mo), respectively (n=4 for each age group). Statistical significance between age groups was analyzed using a *t*-test for human and a one-way ANOVA for mouse, respectively. *P < 0.05, **P < 0.01. N.S.: no significant difference.

To unravel the age-related changes in Cav-1 and collagen levels in the skin, we compared the expression levels of Cav-1 and COL I in the human skin from young- and old-aged groups. To quantify the relative mRNA levels of Cav-1 and COL I, RT-PCR and real-time PCR (RT-PCR/real-time PCR) were performed with the same samples. Western blot analyses could detect two chains of COL I protein bands (α1 and α2 chains). Depending on experimental conditions, 3^rd^ band could be detected as a partial degradation product of COL I. In RT-PCR/real-time PCR and Western blot analyses, Cav-1 was up-regulated, but COL I was down-regulated, in the old-aged group (n=4) compared to the young-aged group (n=4) with statistical significance (Figure 1A-D). These age-dependent changes in Cav-1 and COL I levels in humans prompted a comparison of the levels in skin from mice 6-, 12-, and 18-months-of-age (n=4 for each age group), respectively. The RT-PCR/real-time PCR and Western blot analyses revealed up-regulation of Cav-1 and down-regulation of COL I in skin from 12- and 18-month-old mice, with the changes being more pronounced (but not significantly different) in the latter (Figure 1E-H).

### Negative correlation between Cav-1 and COL I levels is confirmed in Cav-1 knock-down human dermal fibroblasts and Cav-1 knock-out mice

**Figure 2:**
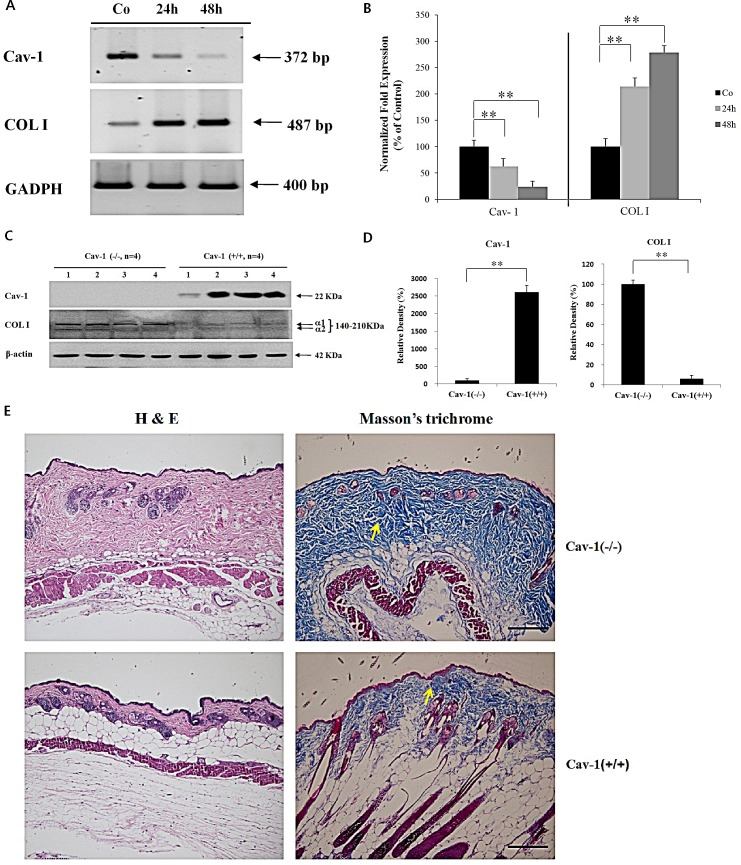
Cav-1 suppression leads to the up-regulated expression of COL I in the HDFs or skin In Cav-1 siRNA transfected HDFs, mRNA expression levels of Cav1 and COL I were checked up to 48 h of post-transfection by (A) RT-PCR and (B) real-time PCR. Statistical analysis from three separate experiments was performed. Co: non-transfected HDFs. In a Cav-1 knock-out experiment, Western blot analysis (C) followed by densitometric analysis (D) for Cav-1 and COL I expression was performed in the Cav-1 (−/−) mice (n=4) and Cav-1 (+/+) littermates (n=4). **P < 0.001. (E) H & E and Masson's trichrome stains were performed for skin samples from Cav-1 (−/−) mice and control Cav-1 (+/+) mice (n=4 for both groups). Scale bar=100 μm for all panels.

Transfection with the Cav-1 small interfering (si) RNA induced marked suppression of Cav-1 mRNA expression up to 48 h after transfection, compared to non-transfected control human dermal fibroblasts (HDFs). Under the same condition, COL I was up-regulated in the Cav-1 siRNA transfected HDFs, showing the negative correlation between Cav-1 and COL I in HDFs (Figures 2A and B). Next, Cav-1 knock-out experiment was performed to confirm the result of Cav-1 knock-down experiment. Cav-1 expression was completely abolished, but COL I level was markedly up-regulated, in Cav-1 (−/−) knock-out mice (n=4) compared to Cav-1 (+/+) littermates (n=4) with a statistical significance (*P* < 0.001) (Figure 2C and D). A marked histological increase of dermal thickness in H & E stained samples (Figure 2E) and up-regulated collagen expression from Masson's trichrome stain (Figure 2E, arrow) were observed in the dermis of Cav-1 (−/−) knock-out mice (n=4 for both groups).

### MβCD up-regulates COL I expression and inhibits Cav-1 expression in the skin of hairless mice

**Figure 3:**
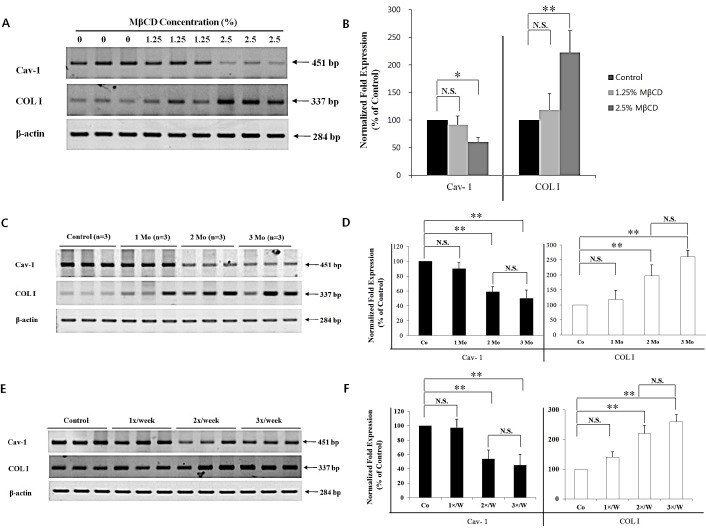
MβCD-induced COL I expression is dependent partly on injection-dose, injection-duration, and injection-frequency in the skin of hairless mice The following results represent three repetitive experiments. RT-PCR (A, C, E) and real-time PCR (B, D, F) were performed with the same samples. Effect of (A, B) injection-dose, (C, D) injection-duration, and (E, F) injection-frequency (n=3 for each group) of MβCD on mRNA expression levels of Cav-1 and COL I in the mouse skin was depicted. Co: saline-injected mice. *P < 0.05, **P < 0.01. N.S.: no significant difference.

The finding of a negative correlation between Cav-1 and COL I levels in chronologically-aged skin prompted the examination of whether COL I expression could be up-regulated *in vivo* by MBCD-mediated inhibition of Cav-1 expression. Twelve-month-old mice were used, based on the results shown in Figure 1. To test the dose-dependency of MBCD activity, hairless mice were divided into two dose-groups of 1.25% MβCD and 2.5% MβCD, based on preliminary optimal dose studies (data not shown), with twice-weekly injections for two months. Determination of mRNA levels revealed that 2.5% MβCD was more potent than 1.25% MβCD in modulating activities of Cav-1 and COL I expression (n=3 for each group) (Figure 3A and B). Control mice (0) were injected with saline instead of MβCD.

**Figure 4:**
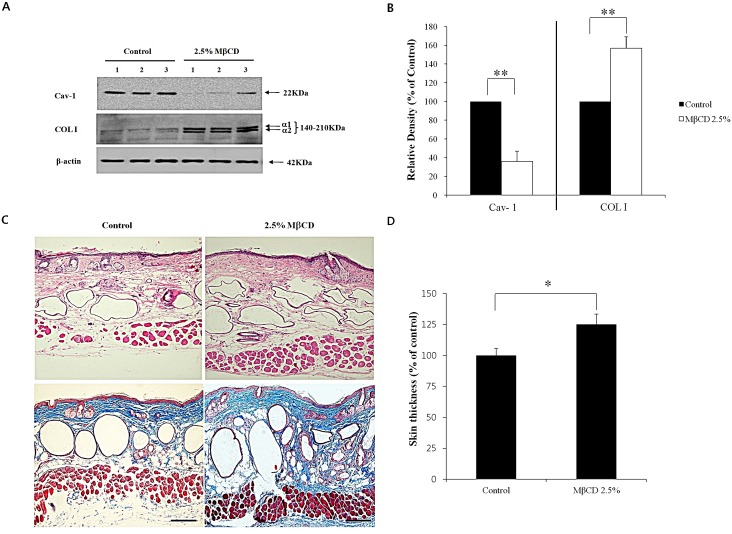
Anti-aging activity of MβCD in the skin of hairless mice (A) Western blot analysis followed by densitometric analysis (B) for Cav-1 and COL I was performed in 2.5% MβCD-injected mice (n=3) twice-weekly for 2 months and saline-injected mice (control, n=3) to verify the results of mRNA experiments. (C) H & E (upper panel) and Masson's trichrome (lower panel) stains for skin samples were performed from 2.5% MβCD-injected and control groups (n=3 for both groups). Bar = 20 μm. (D) Semi-quantitative measurement of skin thickness was performed in MβCD-injected (n=3) and control mice (n=3) groups. Result represents the relative thickness of MβCD-injected mice in comparison with control group (100%). *P < 0.05, **P < 0.01.

To find the optimal COL I-up-regulating activity of 2.5% MβCD, different experimental conditions of administration were tested by adjusting the duration or frequency of injection. For duration testing, mice were injected twice weekly for 1-3 months. Modulation of Cav-1 and COL I mRNA was more prominent in the 2.5% MβCD-injected group for 2-3 months, compared to control or 2.5% MβCD-injected group for 1 month (n= 3 for each group). There was no significant difference between two months and three months of injection-duration in terms of Cav-1 and COL I levels (Figure 3C and D). Next, to ascertain the optimal injection-frequency, mice were divided into three groups, which were injected with 2.5% MβCD once a week (1x/W), twice a week (2x/W), and three times a week (3x/W) for two months. Modulation of Cav-1 and COL I mRNA was more remarkable with 2-3 injections each week (n=3 for each group) (Figure 3E and F). There was no significant difference between injection frequencies of two and three times weekly. Next, down-regulated production of Cav-1 protein and up-regulated production of COL I protein were evident, supporting the mRNA results (Figure 4A and B). Marked histologic increase of dermal thickness was observed in mice injected with 2.5% MβCD for two months, compared to saline-injected control (n=3 for both groups, H & E stain) (Figure 4C, upper panel). The dermal layer was filled with blue staining materials corresponding to collagens (n=3 for both groups, Masson's trichrome stain) (Figure 4C, lower panel). In measuring skin thickness by a dial thickness gauge, mice injected twice-weekly with 2.5% MβCD for two months displayed an 25% increase in skin thickness (125.0 ± 6.3%), compared to control mice (100.0 ± 10.4%) (n=3 for each group, *P* < 0.05) (Figure 4D). The results indicated that twice-weekly intra-dermal injection of 2.5% MβCD for two months produced the optimal conditions as an anti-aging activity of MβCD.

### MβCD-induced COL I expression is skin-specific without adverse reactions

**Figure 5:**
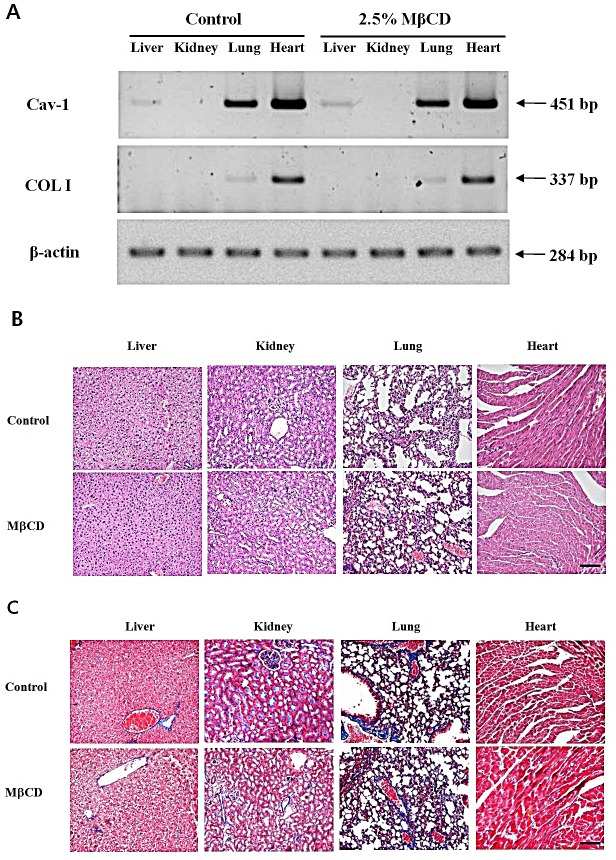
Intra-dermal injection of MβCD does not modulate expression levels of Cav-1 and COL I in the systemic organs of hairless mice (A) RT-PCR for Cav-1 and COL I were performed with total RNA extracts from the tissue samples of MβCD-injected and saline-injected control mice. (B) Histological findings of formalin-fixed, H & E stained skin tissues from 2.5% MβCD- or saline-injected mice (Control) for 3 months. (C) Masson's trichrome stain for collagen expression level in the 2.5% MβCD- and saline-injected control mice. The pictures represent typical results of mice from control and MβCD-injected groups (n= 4 each group). Bar = 20 μm.

To rule out the possibility that intra-dermal injection of MβCD could deleteriously modulate collagen levels in systemic organs, we checked expression levels of Cav-1 and COL I in major internal organs of mice injected twice-weekly with 2.5% MβCD for three months (n=4). Control mice were injected with sterile saline (n=4). MβCD did not modulate mRNA levels of Cav-1 and COL I in liver, kidney, lung, and heart (Figure 5A). In a histological study, four MβCD-injected mice showed no abnormal tissue fibrosis from H & E stain (Figure 5B) and no increase of collagen expression from Masson's trichrome stain (Figure 5C). There was no change in skin texture suggesting skin fibrosis, showing as a velvet as normal skin upon palpation in MβCD-injected mice.

### MβCD up-regulates COL I in HDFs

**Figure 6:**
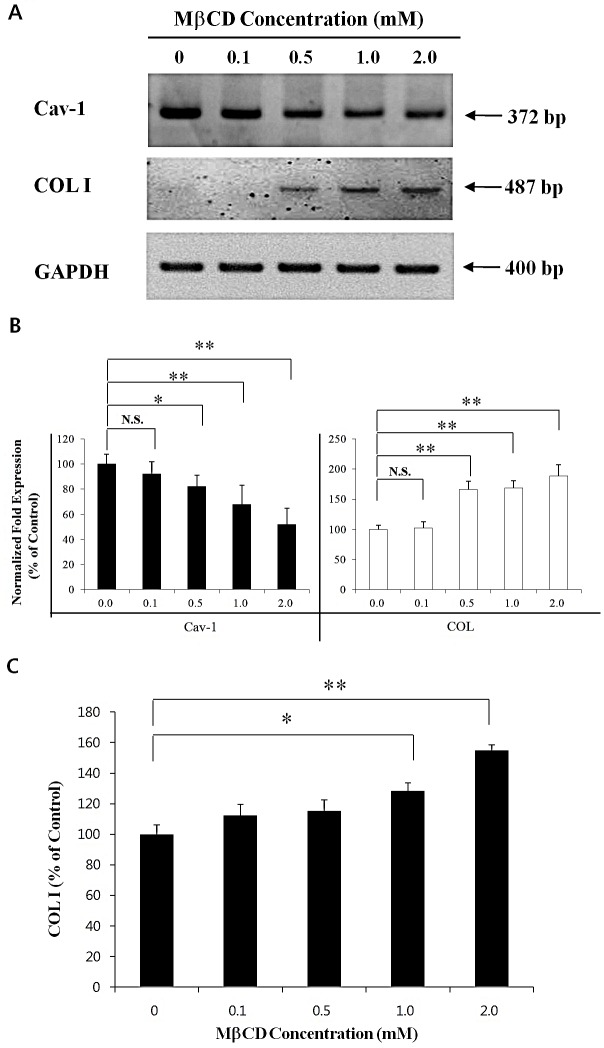
MβCD up-regulates COL I expression in HDFs HDFs were treated with different concentrations of MβCD (0.1-2.0 mM) for 24 h, and total RNAs were prepared. (A) RT-PCR and (B) real-time PCR for Cav-1 and COL I in relation with MβCD concentrations were performed. (C) Enzyme immunoassay to measure COL I protein levels was performed in HDFs, which were treated with different concentrations of MβCD (0.1-2.0 mM) for 24 h. Densitometric analysis was performed from 3 repetitive experiments. *P < 0.05, **P < 0.01. N.S.: no significant difference.

Next, HDFs were treated with different concentrations (0.1-2.0 mM) of MβCD for 24 h to verify the COL I-up-regulating activity of MβCD in mouse skin. RT-PCR/real-time PCR revealed up-regulated COL I and down-regulated Cav-1 by MβCD treatment at 0.5-2.0 mM concentrations (Figures 6A and B). In enzyme immunoassays for COL I, MβCD was revealed to up-regulate COL I protein levels with a statistical significance at 1.0-2.0 mM of MβCD (Figure 6C).

### MβCD-induced anti-aging activity persists for several months after last injection in mouse skin

**Figure 7:**
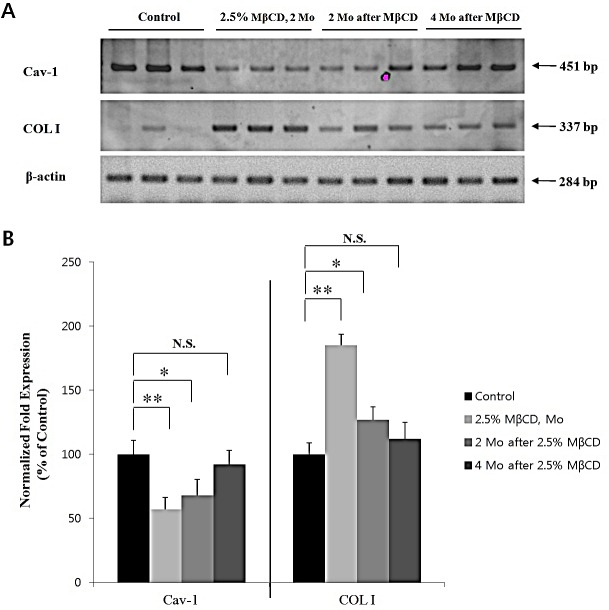
MβCD-induced up-regulation of COL I is maintained for several months in mouse skin After last injection of 2.5% MβCD injection for 2 months, Cav-1 and COL I expression levels in the skin of hairless mice (2.5% MβCD, 2 Mo, n=3) were checked by (A) RT-PCR and (B) real-time PCR. Also, Cav-1 and COL I levels were checked at time-lapse of 2 months (2 Mo after MβCD, n=3) and 4 months (4 Mo after MβCD, n=3) after the last injection of 2.5% MβCD, respectively. Statistical significance was analyzed using a one-way ANOVA, followed by post-hoc comparisons of group means using Tukey's test. *P < 0.05, **P < 0.01. N.S.: no significant difference. Control indicates the saline-injected mice, instead of 2.5% MβCD.

From the above results, MβCD can be developed a novel anti-aging agent by up-regulating COL I level in chronologically-aged skin. Thus, we tested the durability of COL I-up-regulating activity as well as the possibility of irreversible skin fibrosis, in MβCD-injected skin. To test the durability of COL I-up-regulating activity of MβCD, 2.5% MβCD was intra-dermally injected twice-weekly into mouse skin (n=3) for two months, and the mice were kept for another two and four months after the last injection. RT-PCR/real-time PCR demonstrated that down-regulated Cav-1 expression as well as up-regulated COL I expression were induced by MβCD injection for two months. The MβCD-modulated Cav-1 and COL I expressions were also observed at two months after the last injection, but became less prominent as time lapsed after the last injection, ruling out the possibility of irreversible skin fibrosis (Figures 7A and B).

## DISCUSSION

This study demonstrates that decreased COL I level in chronologically-aged skin is closely related with elevated Cav-1 level. There was a negative correlation between expression levels of Cav-1 and COL I in chronologically-aged human and mouse skin *in vivo*. COL I expression could be artificially up-regulated in Cav-1 siRNA transfected HDFs or Cav-1 knock-out mice, which supports the previous reports on knock-down or knock-out experiments [10, 20]. These results support the notion that Cav-1 plays a brake role in COL I expression in the skin. Consistently, Cav-1 was reported to be related with cellular senescence of lung fibroblasts [7].

Skin aging is characterized by the gradual thinning due to decreased total volume of the skin, and age-dependent change of skin volume is mainly determined by collagen levels in the dermis. In general, collagen volume decreases around 30-years-of-age in the dermis of humans. Therefore, anti-aging techniques should be focused on enhancing skin volume by up-regulating collagen expression in the dermis. In this study, MβCD has a potential to be developed as a novel anti-aging agent by up-regulating COL I expression level in HDFs or mouse skin. The optimal COL I-enhancing activity of MβCD was obtained by intra-dermal injection of 2.5% MβCD twice weekly for at least two months in mice.

A variety of approaches to improve skin texture in aged skin have been continuously developed. Replacement of deficient collagens in chronologically-aged skin with mammalian collagen products or synthetic collagen-mimic agents has been applied, but unfortunately, this results in allergic reaction due to the non-human materials [21, 22]. Hyaluronic acid (HA) dermal fillers have been used for the aged skin, as they are biocompatible and safe, despite being of non-biological origin. There are several forms of HA dermal fillers depending on cross-linkers for HA to prolong the durability of the filler [23], but they also have limits for long-term maintenance as anti-aging agents. Considering the average life-expectancy of mice (2 years) compared to humans (80 years), MβCD is expected to be a promising anti-agent agent with long-durability, judging from our result that MβCD persists anti-aging activity at least for two months in mice. As a potential anti-aging agent, MβCD might be a more ideal agent with several advantages. First, collagen synthesis can be stimulated in the skin by inhibiting the action of Cav-1, which is highly up-regulated in the chronologically-aged, normal human skin. Second, foreign body reactions that are frequently related with non-human collagens or fillers can be avoided. Third, collagen (or HA) degradation by collagenases (or hyaluronidases), which could be induced by artificial collagens or HA dermal fillers, is not induced by MβCD. Fourth, collagen expression level in the skin can be controlled by adjusting injection-dose, injection-frequency, and injection-duration of MβCD.

CDs are relatively safe molecules for the skin and systemic organs [17, 18, 24]. We did not observe any remarkable adverse reactions including fibrosis in the skin or systemic organs by intra-dermal injection of MβCD. Considering that the average life expectancy of hairless mice is approximately two years, three months of MβCD injection may be long enough to verify the long-term safety of MβCD in the body. The 2.5% MβCD used in this study represents approximately 80 mg/kg/day, which is considered to be fairly low dose in comparing with another study [18]. In a standard battery of genotoxicity tests, none of the tested CDs were genotoxic or mutagenic [18]. Our results together with the safety profiles of studied CDs indicate that MβCD as a CD derivative might be very safe as a new anti-aging agent in human skin.

The COL I-up-regulating activity of MβCD exerted as partly dose-dependent manners in relation with the injection dose, duration, and frequency. However, the MβCD-induced COL I-up-regulating activity was not further enhanced by injection more than twice per week or duration of injection longer than two months, suggesting that the MβCD activity reaches a plateau in the skin. Similar findings were observed in age-dependent changes of Cav-1 and COL I levels, in that there were no significant changes in Cav-1 and COL I levels of mouse skin between 12- and 18-months-of-age. We expect that COL I-up-regulating activity of MβCD is more physiological than existing available anti-aging techniques, such as administering fillers or botulinum toxin [23, 25]. Development of anti-agent agents by modulating collagen expression should be done with caution, however, since the same mechanism might play a crucial role in skin fibrosis [26]. Fortunately, MβCD is supposed not to induce skin fibrosis or fibrosis of major systemic organs of heart, lung, liver, and kidney from our RT-PCR and H & E results. Physical examination demonstrated that skin texture of MβCD-injected mice was normally velvety-smooth, implying that skin fibrosis might not be induced by MβCD-injection. Furthermore, our result that the COL I-up-regulating activity of MβCD in mouse skin was decreased as time lapsed after last injection of MβCD verifies that the COL I-enhancing activity of MβCD is reversible.

In conclusion, MβCD is found to be a safe COL I-up-regulating agent without serious adverse reactions including skin fibrosis. Our results suggest that MβCD can be developed as a novel anti-aging agent in the skin. Further studies on long-term safety as well as durability of anti-aging activity of MβCD in humans are warranted. Inhibition of Cav-1 expression through anti-Cav-1 decoys or skin-permeable anti-Cav-1 peptides instead of chemical inhibitors can be a new strategy in developing anti-aging agents in the skin [17].

## MATERIALS AND METHODS

### Experiments in humans and mice

Human samples from the abdomen were obtained after informed consent according to the guidelines of the local ethics committee of our hospital and the Declaration of Helsinki Principles (IRB No.: 1-2009-11-136). For the old-aged skin, samples were obtained from two individuals in their 70s and two individuals in their 80s. For the young-aged skin, four teen-agers were enrolled. For mouse experiments, official permission to handle mice was obtained from the animal committee of our university. SKH1 hairless mice were purchased from the Orient Bio (Iksan, Korea). For experiments to study age-dependent changes in Cav-1 and COL I levels, mice were bred up to 6-, 12-, and 18-months-of-age. Anti-aging activity of MβCD was studied in mice of 12-months-of-age. For knock-out experiments, female C57BL mice target-mutated for Cav-1 were purchased from the Jackson Laboratory (Bar Harbor, ME).

### Preparation of MβCD solution

MβCD was purchased from Tokyo Chemical Industry (Tokyo, Japan). MβCD solution was prepared by dissolving the chemical in sterile saline and injected into the dermis of hairless mice using a 27-gauge needle to deliver 100 μl of MβCD solution per injection (daily dose: 1.25-5.0 mg per mouse).

### Cell culture

HDFs (Cascade Biologics, Portland, OR) were sub-cultured at a ratio of 1:4 in 10-cm plates with Dulbecco's modified Eagle's medium (DMEM) supplemented with 10% fetal bovine serum and antibiotics.

### Preparation of protein and total RNA extracts from the skin and HDFs

The excised skin samples were immediately frozen by liquid nitrogen and stored at −70°C until required for experiments. Protein extracts and total RNAs from the skin samples and HDFs were prepared as described before [27].

### Western blotting for Cav-1 and COL I

Western blotting was performed as described previously [28]. The protein bands were probed with the antibodies (human and mouse) to anti-Cav-1 (ab28947, 1:1,000; Abcam, Cambridge, MA), COL1A2 (Y-18, 1:200; Santa Cruz Biotechnology, Santa Cruz, CA), and anti-β-actin (ab-6276, 1:5,000; Abcam) overnight at 4^o^C. Densitometric analyses were performed by using the Multi Gauge V3.0 software (Fujifilm, Tokyo, Japan).

### RT-PCR/real-time PCR

PCR reactions were performed using the following primers: mouse Cav1 (forward: 5′-aaaaggaggctgctaaaccg-3′; reverse: 5′-tatcggcaagactgaaggag-3′); human CAV1 (forward: 5′-gttcccttaaagcacagccc-3′; reverse: 5′-gacagcaagcggtaaaacca-3′); mouse Col I (forward: 5′-ctttgcttcccagatgtcctat-3′; reverse: 5′-cggtgtcccttcattccag-3′); human COL I (forward: 5′-atgatgagaaatcaaccgga-3′; reverse: 5′-ccagtagcaccatcatttcc-3′); mouse β-actin (forward:5′-tcatgaagtgagacgttgacatccgt-3′; reverse: 5′-cctagaagcacttgcggtgcacgatg-3′); and human glyceraldehyde 3-phosphate dehydrogenase (GAPDH; forward: 5′-gtcttcaccaccatggagaaggc-3′; reverse: 5′-cggaaggccatgccagtgagctt-3′). Annealing temperatures were 55^o^C for human COL I; 58°C for mouse Cav1, mouse Col I and mouse β-actin; and 60^o^C for human CAV1 and human GAPDH. Expression levels were normalized to an endogenous β-actin or GAPDH levels. For experiments to measure mRNA levels, RT-PCR/real-time PCRs were performed with the same primer sets for target genes. Real-time PCR was done in triplicate with the HOT FIREPol EvaGreen® qPCR Mix Plus (Solis BioDyne, Tartu, Estonia) using a RotorGene 3000 (Corbett Research, Cambridge, U.K.). The thermal cycling condition was 15 min at 95^o^C, followed by 40 cycles of 95^o^C for 10 sec, 55-60^o^C for 20 sec, and 72^o^C for 30 sec. The relative abundance of a given transcript was estimated using the 2^−ΔΔCt^ method, following normalization to β-actin or GAPDH.

### Knock-down of Cav-1 by siRNA transfection

Transfection was carried using Oligofectamine (Invitrogen, Carlsbad, CA) transfection reagent. Small interfering RNA (siRNA) was designed against the coding sequence of Cav-1 cDNA using software by scitools (Integrated DNA Technologies, Coralville, IA). CAV-1 siRNA sequence primers were forward, 5′-GCAUUAAGAGCUUCCUGAUUGAGAT-3′ and reverse, 5′-AUCUCAAUCAGGAAGCUCUUAAUGCAU-3′. Cells in 6-well plates at 50-60% confluency were incubated with 3 ml of culture media lacking antibiotics and containing 30 μl of Oligofectamine in the absence and presence (30 μM) of Cav-1 siRNA. After 6 h, the cells were washed in 1X PBS, and were cultured in new DMEM lacking fetal bovine serum. Cells were incubated at 37^o^C in a CO_2_ incubator for 48 h until ready to assay for gene knockdown.

### Quantitative measurement of COL I by ELISA

Expression levels of COL I protein were determined by the commercially available Procollagen type I C-peptide enzyme immunoassay kit (Takara, Shiga, Japan), and absorbance was determined at 450 nm with a microplate reader (Molecular Devices, Sunnyvale, CA).

### Measurement of skin thickness

Skin thickness was measured with a dial thickness gauge (Peacock, Scotts Valley, CA). After hairless mouse was killed by neck dissection, skin flap containing the epidermis and dermis was immediately obtained by dissecting skin layer from the underlying muscle with iris scissors. Then, the skin flap was inserted between the contact points of dial thickness gauge. For accurate measurement, we prepared relatively large-sized skin flap, measuring at least 3 cm in diameter.

### Statistics

Data are expressed as mean ± standard deviation (SD). Statistical analyses were performed by independent *t*-test or by one-way analysis of variance (ANOVA) with post hoc Tukey's test using SPSS v13.0 (SPSS, Chicago, IL), when multiple comparisons were made. *P* < 0.05 was considered indicative of a statistically significant difference.

## Abbreviations

Cav-1: caveolin-1
COL I: collagen I
HDFs: human dermal fibroblasts
MβCD: Methyl-β-cyclodextrin
